# Confined small-sized cobalt catalysts stimulate carbon-chain growth reversely by modifying ASF law of Fischer–Tropsch synthesis

**DOI:** 10.1038/s41467-018-05755-8

**Published:** 2018-08-14

**Authors:** Qingpeng Cheng, Ye Tian, Shuaishuai Lyu, Na Zhao, Kui Ma, Tong Ding, Zheng Jiang, Lihua Wang, Jing Zhang, Lirong Zheng, Fei Gao, Lin Dong, Noritatsu Tsubaki, Xingang Li

**Affiliations:** 1 Collaborative Innovation Center of Chemical Science and Engineering (Tianjin), Tianjin Key Laboratory of Applied Catalysis Science and Technology, School of Chemical Engineering and Technology, Tianjin University, 300072 Tianjin, China; 2 Shanghai Synchrotron Radiation Facility, Shanghai Institute of Applied Physics, Chinese Academy of Sciences, 201800 Shanghai, China; 3 Beijing Synchrotron Radiation Facility, Institute of High Energy Physics, Chinese Academy of Sciences, 100049 Beijing, China; 4 Jiangsu Key Laboratory of Vehicle Emissions Control, Center of Modern Analysis, Nanjing University, 21009 Nanjing, China; 5 Department of Applied Chemistry, School of Engineering, University of Toyama, Gofuku 3190, Toyama, 930-8555 Japan

## Abstract

Fischer–Tropsch synthesis (FTS) is a promising technology to convert syngas derived from non-petroleum-based resources to valuable chemicals or fuels. Selectively producing target products will bring great economic benefits, but unfortunately it is theoretically limited by Anderson–Schulz–Flory (ASF) law. Herein, we synthesize size-uniformed cobalt nanocrystals embedded into mesoporous SiO_2_ supports, which is likely the structure of water-melon seeds inside pulps. We successfully tune the selectivity of products from diesel-range hydrocarbons (66.2%) to gasoline-range hydrocarbons (62.4%) by controlling the crystallite sizes of confined cobalt from 7.2 to 11.4 nm, and modify the ASF law. Generally, larger Co crystallites increase carbon-chain growth, producing heavier hydrocarbons. But here, we interestingly observe a reverse phenomenon: the uniformly small-sized cobalt crystallites can strongly adsorb active C* species, and the confined structure will inhibit aggregation of cobalt crystallites and escape of reaction intermediates in FTS, inducing the higher selectivity towards heavier hydrocarbons.

## Abstract (web summary)

Fischer–Tropsch synthesis (FTS) is theoretically limited by Anderson–Schulz–Flory (ASF) law. Here, the authors successfully tune the selectivity of products from diesel-range hydrocarbons to gasoline-range hydrocarbons in FTS by controlling the crystallite sizes of confined cobalt, and modify the ASF law.

## Introduction

Concerns over global oil depletion bring urgent demands to find alternative feedstocks to produce important petrochemicals and fuels. Fischer–Tropsch synthesis (FTS) can synthesize clean liquid fuels via catalytic polymerization of syngas (CO and H_2_)^1–3^. In order to obtain the high yield of the target product in FTS, it is necessary to control the selectivity of the corresponding fractions. Unfortunately, over conventional FTS catalysts, the selectivity of products follows the Anderson–Schulz–Flory (ASF) law, which is unselective for the target products^2^. For example, the maximum selectivities to C_5_­–C_11_ (gasoline-range) and C_10_–C_20_ (diesel-range) hydrocarbons are ∼45% and ∼39%, respectively. Conventionally, the FTS products are subjected to further hydro-treatment to improve the selectivity of liquid fuels^4^. The direct production of target-range liquid fuels will be more efficient in energy and economically effective than the multiple-stages process. It is urgent to design high-performance FTS catalysts with a high selectivity to target products^4–6^.

Metallic cobalt, ruthenium, and iron are conventional FTS catalysts. Cobalt-based catalysts have the advantages of the low activity in water gas shift reaction, and the high catalytic activity and stability in FTS^6,7^. The crystallite sizes of cobalt can dramatically affect the catalytic activity and selectivity for FTS^7–11^. De Jong’s group^8,9^ reported a non-classical structure sensitivity for the Co/CNFs catalysts. The turnover frequency (TOF) values of the catalysts continuously increased with the enlarged crystallite sizes of cobalt, and then became constant when the crystallite sizes were larger than 6–8 nm. They also found that the C_5_^+^ selectivity of the products increased from 51 to 85%, when the crystallite sizes of cobalt increased from 2.6 to 16 nm. Although this view is generally accepted, it is still under debate. Borg et al.^10^ and Holmen’s group^11^ reported that the C_5_^+^ selectivity showed a volcano-like variation via the enlarged sizes of the cobalt crystallites. In their work, the C_5_^+^ selectivity increased with the enlarged crystallite sizes of cobalt smaller than ~8 nm and reached the maximum value, and then it dropped with the further enlarged cobalt crystallites over the Co/Al_2_O_3_ catalysts^10,11^. Nevertheless, Iglesia^12^ claimed that the intrinsic reaction rate, as well as the chain-growth of products, did not depend on the crystallite sizes of cobalt in FTS. Thus, it is important to clarify the function of the crystallite sizes of cobalt on the selectivity of products to rationally design and synthesize highly selective FTS catalysts towards specific products.

Researchers have controlled the crystallite sizes of cobalt by different preparation routes, such as incipient wetness impregnation^11,12^, electrostatic adsorption^13^, and homogeneous deposition-precipitation^14^. In general, cobalt will gradually aggregate with proceeding of FTS reactions, which probably brings conflict results on function of crystallite sizes of cobalt on selectivity of products. Confinement structure of catalysts is one of appropriate strategies to stabilize and immobilize metallic crystallites. Somorjai’s group^15^ reported that inorganic silica shells encaged Pt crystallite exhibited high catalytic activities for ethylene hydrogenation and CO oxidation because of restricting sintering of Pt crystallites at high temperatures. Bao’s group^3^ also reported that encapsulation of iron species inside carbon nanotubes could effectively inhibit aggregation of iron crystallites, and enhance selectivities towards heavy hydrocarbons ascribed to inhibition of diffusion of reaction intermediates.

Herein, we synthesize the size-uniformed Co_3_O_4_ nanocrystals, which are then embedded into the mesoporous SiO_2_ support to avoid aggregation in FTS. Surprisingly, we can successfully tune the selectivity of the FTS products from the diesel fraction (66.2%) to the gasoline fraction (62.4%) by controlling the crystallite sizes from 7.2 to 11.4 nm of the confined cobalt catalysts. It should be noticed that the small-sized cobalt crystallites with the confined structure interestingly give the large chain growth probability (*α*) and the high selectivity towards heavy hydrocarbons.

## Results

### Catalyst synthesis process and structure

**Fig. 1:**
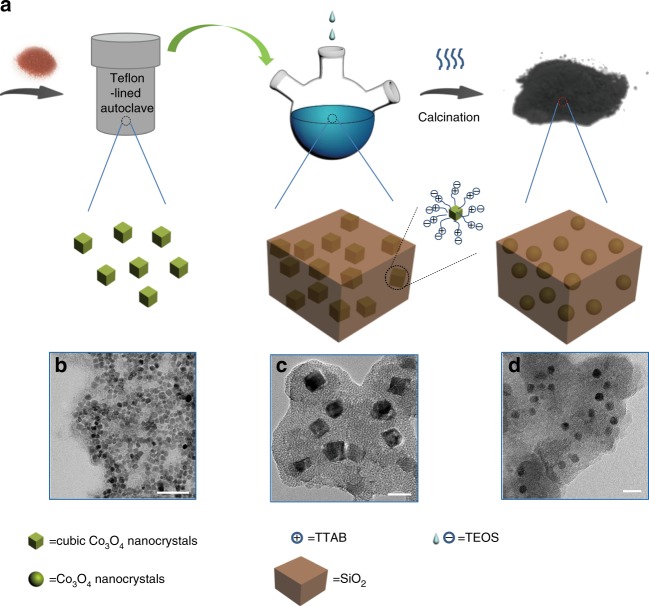
Synthesis of the Cat-*x*h catalysts. **a** Schematic illustration of the synthesis of the Co_3_O_4_ nanocrystals with the narrow size distribution and the preparation of the Cat-*x*h catalysts with the uniform size distribution. **b**–**d** TEM images of the materials containing Co_3_O_4_ hydrothermal-synthesized for 8 h. **b** TTAB-capped Co_3_O_4_ nanocrystals. **c** Co_3_O_4_-TTAB-silica nanocomposite. **d** Cat-8h. Scale bars: **b** 50 nm; **c** 10 nm; **d** 20 nm

Figure 1a describes the catalyst synthesis process. We synthesized the Co_3_O_4_ nanocrystals using tetradecyltrimethlammonium bromide (TTAB) as the capping agent with a facile hydrothermal process. Varying the hydrothermal duration can control the sizes of the Co_3_O_4_ nanocrystals. Thereafter, we utilized the silica-TTAB layer to wrap the TTAB-capped Co_3_O_4_ nanocrystals through an electrostatic interaction between the cationic (TTAB) and anionic (tetraethyl orthosilicate (TEOS)) species. After calcination of the above powder in air to remove TTAB, we successfully achieved the final catalyst, which was denoted as Cat-*x*h (*x* = 4, 8, 12), where ‘*x*h’ was the hydrothermal synthesis period of the Co_3_O_4_ nanocrystals. Figures 1b, c show the transmission electron microscopy (TEM) images for TTAB-capped Co_3_O_4_ and Co_3_O_4_-TTAB-silica, respectively. The TTAB-capped Co_3_O_4_ nanocrystals in Fig. 1b and Supplementary Fig. 1 mainly exist in cubic shape and exhibit a uniform size distribution. The results of TEM (Supplementary Fig. 1) and X-ray diffraction (XRD; Supplementary Fig. 2) of the TTAB-capped Co_3_O_4_ nanocrystals reveal that the sizes of the Co_3_O_4_ nanocrystals increase with the prolonged hydrothermal duration. Figure 1c shows the assembled structure of the Co_3_O_4_-TTAB-silica nanocomposites.

After calcination of the Co_3_O_4_-TTAB-silica nanocomposites, the cubic Co_3_O_4_ nanocrystals changed to the spheric shape (Fig. 1d). For the Cat-*x*h catalysts (Table 1, Supplementary Fig. 3 and Supplementary Table 1), capping of silica did not change the original sizes of the Co_3_O_4_ nanocrystals. Table 1 gives the textural properties of these catalysts evaluated by N_2_ physisorption. The results of nitrogen adsorption-desorption isotherms reveal that the Cat-*x*h catalysts (Supplementary Fig. 4) are mesoporous^15^ with the surface areas of ~320 m^2^ g^−1^. Summarily, our Cat-*x*h catalysts show a water-melon-like structure, where water-melon seeds (cobalt nanocrystals) are embedded inside pulps (silica bulk matrix).

**Table 1:** Chemical and physical properties of the catalysts

Catalysts	Metal content		Co / Si in molar ratio (%)^b^	Surface areas (m^2^ g^−1^)	Pore volume (cm^3^ g^−1^)	Metal sizes							
	Co (wt. %)^a^	Co (wt. %)^b^				XRD		H_2_-Chemisorption				TEM	
						*d*(Co_3_O_4_)_X_ (nm)^c^	*d*(Co^0^)_X_ (nm)^d^	H_2_-uptake (μmol g^−1^)	*f* (%)^e^	*D* (%)^f^	*d*(Co^0^)_H_ (nm)^g^	*d*(Co^0^)_T_ (nm)^h^	*σ* (nm)^i^
Cat-12h	13.9	8.3	14.1	331	0.23	12.8	9.6	80	75.2	9.1	10.6	11.4	1.7
Cat-8h	14.5	6.9	11.6	329	0.22	11.0	8.3	95	71.5	10.8	8.9	9.1	1.5
Cat-4h	15.3	5.8	9.6	312	0.23	8.9	6.7	118	68.9	13.2	7.3	7.2	1.4
Cat-IM	16.3	12.6	22.5	165	0.48	13.1	9.8	88	81.6	7.8	12.3	14.3	2.8

^a^From ICP-MS

^b^From XPS

^c^Calculated using the Scherrer’s equation. The suffix X denoted XRD

^d^Calculated applying the molar volume correction: $d({\mathrm{Co}}^0)_{\mathrm{X}} = \frac{3}{4}d({\mathrm{Co}}_3{\mathrm{O}}_4)_{\mathrm{X}}$. The suffix X denoted XRD

^e^Reduction degree

^f^Metal dispersion

^g^The suffix H standing for H_2_-chemisorption

^h^From TEM images. The suffix T standing for TEM

^i^Standard deviation for the crystallite size distribution

**Fig. 2:**
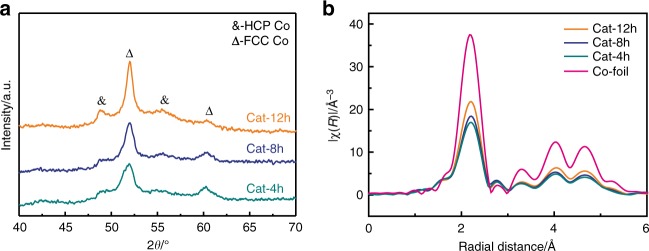
Structure of the reduced catalysts. **a** XRD patterns of the reduced Cat-*x*h catalysts. The crystallite sizes of metallic cobalt in the reduced catalysts are estimated to be 10.2, 8.2, 6.5 nm based on the Scherrer’s equation. **b** RDFs spectra of the reduced catalysts and the reference Co foil

Figure 2a shows the XRD patterns of the reduced Cat-*x*h catalysts. We observed two types of the metallic cobalt phases including the cubic phase with the strong diffraction peaks (face-centered cubic (FCC); JCPDS 15-0806) and the hexagonal phase with the weak diffraction peaks (hexagonal close-packed (HCP); JCPDS 05-0727)^16^. The crystallite sizes of metallic cobalt have a similar tendency with the above mentioned Co_3_O_4_ nanocrystals. Figure 2b shows the Co K*-*edge radial distribution functions (RDFs) of the reduced Cat-*x*h catalysts and the standard Co-foil. The RDFs of the reduced Cat-*x*h catalysts display the remarkably lower peak amplitudes than that of the Co foil, indicating the formers’ lower coordination numbers. Supplementary Table 2 gives the simulated data determined from Fig. 2b. The cobalt species in the reduced Cat-*x*h catalysts exist in the metallic state. The Co–Co coordination numbers of the reduced Cat-4h, Cat-8h, and Cat-12h catalysts are 7.3, 8.1, and 9.3, respectively, which are much smaller than that of the Co foil (12.0). These findings suggest that the dispersion of the cobalt species follows the sequence of Cat-4h>Cat-8h>Cat-12h, as well as an inverse sequence of the crystallite sizes.

### Embedment of Co_3_O_4_ nanocrystals

Table 1 gives the metal dispersion (*D*) and mean particle sizes (*d*(Co^0^)_H_) of cobalt determined from the H_2_-chemisorption. In Table 1, the Cat-IM catalyst prepared by a conventional incipient-wetness impregnation method (IM) displays a low metal dispersion (7.8%) with a *d*(Co^0^)_H_ of 12.3 nm. Nevertheless, its *d*(Co^0^)_H_ is substantially larger than the value (*d*(Co^0^)_X_ = 9.8 nm) estimated from the XRD characterization. It suggests that migration and growth of the metallic cobalt take place upon H_2_ reduction of the Cat-IM catalyst, owing to a weak interaction between the cobalt and the support. Nevertheless, the *d*(Co^0^)_H_ values are similar to those derived from the XRD characterization for the reduced Cat-*x*h catalysts.

**Fig. 3:**
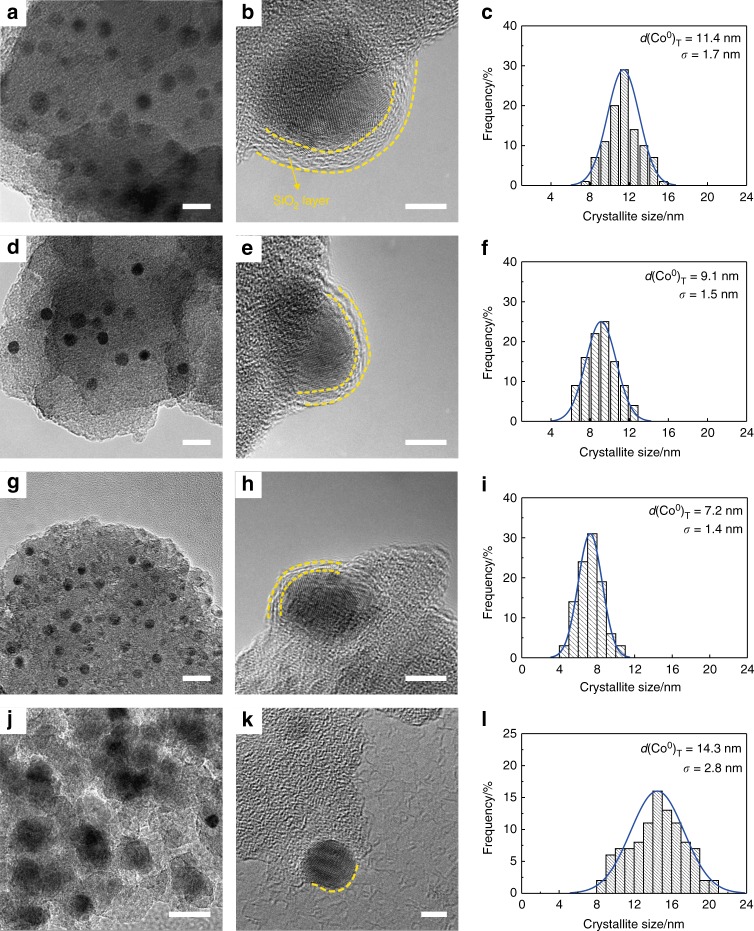
TEM images and corresponding crystallite size distributions of the reduced catalysts. **a**, **d**, **g**, **j** TEM and **b**, **e**, **h**, **k** HRTEM images of the reduced Cat-*x*h catalysts. **c**, **f**, **i**, **l** Corresponding crystallite size distributions of the reduced catalysts. **a**, **b**, **c** Cat-12h, **d**, **e**, **f** Cat-8h, **g**, **h**, **i** Cat-4h, and **j**, **k, l** Cat-IM. Scale bars: **a**, **d**, **g**, **j** 20 nm; **b**, **e**, **h**, **k** 5 nm

Figure 3 shows the TEM images and the corresponding distributions of the crystallite sizes of cobalt of the reduced catalysts. In Fig. 3, the metallic cobalt crystallites are homogeneously dispersed on the reduced Cat-*x*h catalysts with a narrow size distribution. Especially, they are embedded inside the mesoporous silica support in a water-melon-like structure (Fig. 3b, e, h). The embedment strategy has enormous advantage in controlling the size of cobalt than the traditional impregnation method (Supplementary Figs. 5,  6 and Fig. 3g–l). It indicates that the embedded spatial confinement of the Cat-*x*h catalysts can effectively prevent the metal migration and growth. It well explains the little difference between *d*(Co^0^)_H_ and *d*(Co^0^)_X_ for the confined Cat-*x*h catalysts in Table 1.

**Fig. 4:**
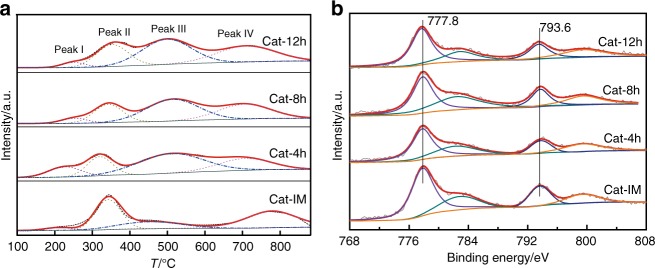
Embedment of Co species. **a** H_2_-TPR profiles of the catalysts. **b** In situ XPS spectra of the catalysts at Co 2*p* core levels

Figure 4a shows the temperature-programmed reduction of H_2_ (H_2_-TPR) profiles of the catalysts. The Peak I and II below 400 °C can be assigned to the reduction of the surface and the bulk of Co_3_O_4_, respectively^17^. The Peak III may be ascribed to the reduction of the cobalt species deeply embedded into silica because of the diffusion limitation of H_2_^6^, and the Peak IV located above 600 °C can be assigned to the reduction of cobalt species strongly interacted with silica^7,18,19^. In Supplementary Table 3, the areas of the Peak III for the Cat-*x*h catalysts follow the sequence of Cat-4h>Cat-8h>Cat-12h, and are much larger than that of the Cat-IM catalyst. It indicates that compared with the Cat-IM catalyst, more cobalt species are deeply embedded into silica on the Cat-*x*h catalysts, further confirming the embedment structure of the Cat-*x*h catalysts.

In addition, we evaluated the composition of the surface atoms of the catalysts using in situ X-ray photoelectron spectroscopy (XPS; Fig. 4b). The values of the Co/Si atomic ratio of the Cat-*x*h catalysts increase from 9.6 to 14.1% with the increase of the crystallite sizes of metallic cobalt (Table 1), but all of them are much lower than that of the Cat-IM (22.5%). In general, the smaller crystallite size of cobalt on the catalysts will expose the more superficial cobalt species. But, the results of in situ XPS break the rule, resulting from the embedment of cobalt into silica for the Cat-*x*h catalysts. Moreover, despite of the high loading (about 15 wt.%) and the excellent dispersion of cobalt, only 5.8–8.3 wt.% of cobalt locates on the surface of the Cat-*x*h catalysts, further certifying the embedment of the cobalt species into the silica support therein.

### Catalytic behaviors in FTS

We evaluated the catalytic activity and selectivity of the Cat-*x*h catalysts in FTS, and Table 2 gives the related data. In Table 2, with the enlargement of the metallic cobalt crystallites the CO conversion slightly increases from 77.0 to 80.6%, meanwhile the TOF value remarkably increases from 3.9 × 10^−2^ to 6.4 × 10^−2^ s^−1^.

**Table 2:** FTS activity data of the catalysts

Catalysts	CO Conversion (%)	TOF (s^−1^)	Selectivity (%)					
			CH_4_	C_2_–C_4_	C_5_^+^	C_5_–C_11_	C_10_–C_20_	C_21_^+^
Cat-12h	80.6	0.064	8.7	11.3	80.0	62.4	23.7	5.7
Cat-8h	78.2	0.049	10.4	14.7	74.9	42.4	39.3	6.4
Cat-4h	77.0	0.039	8.0	7.8	84.2	15.6	66.2	7.8
Cat-IM	84.0	0.065	18.0	14.7	67.3	43.5	29.7	5.1

Note: Reaction condition: *P* = 2 MPa, *T* = 220 °C, W/F = 5.1 g_cat_ h mol^−1^, CO/H_2_ = 1/2

**Fig. 5:**
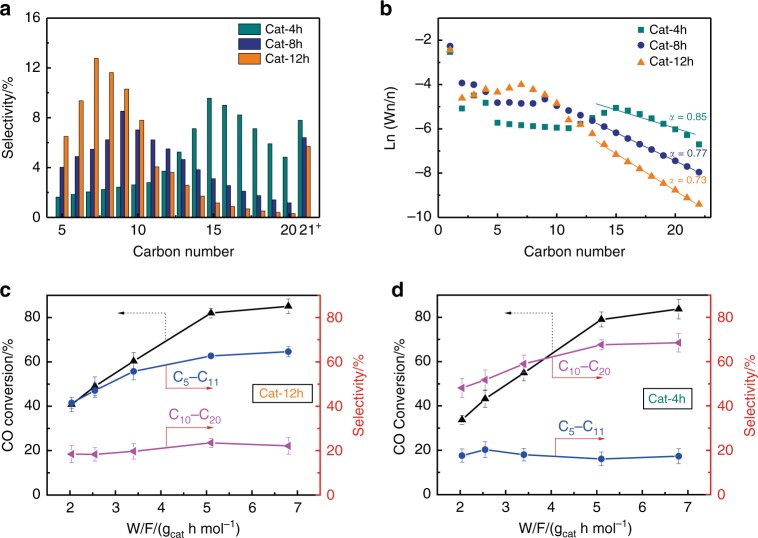
FTS performance of the Cat-*x*h catalysts. **a** Distributions of the C_5_^+^ products of the Cat-*x*h catalysts. **b** ASF distributions for the Cat-*x*h catalysts. Effect of contact time on activity and selectivity of **c** Cat-12h, and **d** Cat-4h. Reaction conditions: *T* = 220 °C, *P* = 2 MPa, W/F = 2.0-6.8 g_cat_ h mol^−1^, CO/H_2_ = 1/2. Error bars indicate s.d. (*n* = 5)

Table 2 and Fig. 5a also provide the FTS product distributions over the Cat-*x*h catalysts. It should be noticed that generally, the references only reported the C_5_^+^ selectivity^7–12^. But for our Cat-*x*h catalysts, no obvious tendency towards the C_5_^+^ selectivity was observed, which varied from ~ 75 to 84%. But, very interestingly, the Cat-12h catalyst exhibits a C_5_–C_11_ (gasoline-range) selectivity of 62.4%; the Cat-8h catalyst exhibits a C_5_–C_11_ selectivity of 42.4% and a C_10_–C_20_ (diesel-range) selectivity of 39.3%; and the Cat-4h catalyst exhibits a C_10_–C_20_ selectivity of 66.2%. Surprisingly, through controlling the crystallite sizes of cobalt and designing of the confined reaction field, we can tune the selectivity of the FTS products from the lower molecular weights, such as C_5_–C_11_, to the higher molecular weights, such as C_10_–C_20_ (Fig. 5a). However, unlike the confined Cat-*x*h catalysts, the Cat-IM-*x*h catalysts prepared by impregnating as-synthesized Co_3_O_4_-*x*h precursors over silica supports do not exhibit a narrow product distribution for either gasoline-range or diesel-range hydrocarbons with the size change of the cobalt particles (Supplementary Table 4 and Supplementary Fig. 7). It indicates the important role of the embedment structure of the Cat-*x*h catalysts on the FTS product selectivity. Figure 5b and Supplementary Fig. 8 show the *α* values of the catalysts. The *α* values of the Cat-*x*h catalysts decrease from 0.85 of the Cat-4h to 0.73 of the Cat-12h with the increase of the crystallite sizes of cobalt. It indicates that the smaller crystallite sizes of the confined cobalt catalyst favor chain growth, resulting in the higher selectivity of heavy hydrocarbons. The results of the repeated experiments on Cat-12h (Supplementary Fig. 9a and Supplementary Table 5) and Cat-4h (Supplementary Fig. 9b and Supplementary Table 6) catalysts prove that our catalysts have good reproducibility.

Generally, textural structures^20–22^, acid properties^23–25^, crystallite sizes of metal species^11,26,27^ and confinement structures^3,6,26^ can affect product distributions in FTS. The textural structures can affect the diffusibility of reactants and products leading to varied activities and selectivities^20–22^. We did not observe an obvious change for the textural structures of the Cat-*x*h catalysts in Table 1. Moreover, we carried out the experiments with the different contact time over the Cat-12h (Fig. 5c) and Cat-4h catalysts (Fig. 5d). With the prolonging of contact time, the Cat-12h and Cat-4h catalysts still kept the high selectivity towards C_5_–C_11_ and C_10_–C_20_, respectively. The acid sites on the catalyst may initiate the secondary cracking, isomerization, and aromatization of the primary FTS products^23–25^. There exist some acidic functional groups, such as hydroxyl and carboxyl, on the surface of our silica support, inducing the acidity of the catalysts. The results (Supplementary Fig. 10) of the NH_3_-temperature programmed desorption and Fourier transform infrared spectroscopy of pyridine adsorption suggest that the acidic features of the Cat-*x*h catalysts are very close. Therefore, the textural structures and acid properties of the Cat-*x*h catalysts did not play a dominant role in determining the selectivity of the FTS products. Catalysts with different metal sizes will expose different surface sites, and a uniform crystallite size here will guarantee the reaction occurring on similar surface sites to produce specific products^26–29^. Indeed, Kang et al.^30^ found that the uniform Fe_5_C_2_-based nanocatalysts presented the selectivity of ~38% towards gasoline-range hydrocarbons. Similarly, our Cat-*x*h catalysts realize the specific product selectivity because of their uniform crystallite size distributions. Spatial confinement structures may affect accessibility of active sites to reaction intermediates, determining varied selectivities^3,5,6,31^. For example, the Cat-12h and Cat-IM catalysts have the similar number of the active sites (Table 1), but the C_5_^+^ selectivity of the former (80.0%) is higher than that of the latter (67.3%) because of the confinement effect. The spatial confinement of the silica prevents the aggregation of the cobalt nanocrystals, and prolongs contact time of the trapped reaction intermediates with the active sites, inducing the growth of the long chain hydrocarbons, beyond the classic ASF model.

In order to further clarify the role of silica confinement, we synthesized additional two reference catalysts of Cat-4h-1 and Cat-4h-2 using the similar preparation method with the Cat-4h catalyst, except that the TEOS/Co molar ratios of the Cat-4h-1, Cat-4h, and Cat-4h-2 were 2, 4, and 8, respectively. Supplementary Fig. 11a-c displays the TEM images of the reduced catalysts. Increasing the TEOS/Co molar ratios will embed cobalt crystallites deeper into the silica bulk matrix^32–34^. Their FTS activity data based on the same cobalt content are given in Supplementary Table 7. Although the depth of the cobalt crystallites embedded into the silica is different, they exhibit the similar catalytic behavior (CO conversion and C_5_^+^ selectivity), and especially the high selectivity towards C_10_–C_20_ products. Probably, it is because that they have the same size of the cobalt crystallites, guaranteed by the confined structure, and thus exhibit the similar product distribution.

**Fig. 6:**
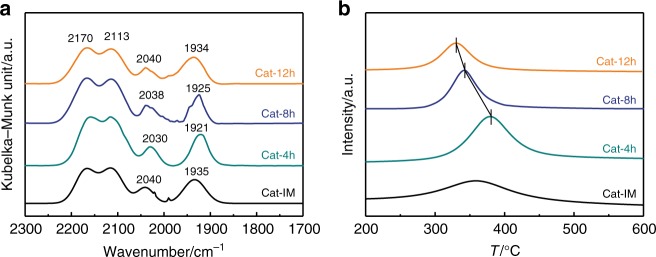
Interaction between CO and the reduced catalysts. **a** In situ DRIFTS spectra of CO adsorption on the reduced catalysts. **b** CO-TPD/MS profiles of the reduced catalysts

We conducted the experiments of in situ diffuse reflectance infrared Fourier transform spectroscopy (DRIFTS) with adsorption of CO to illuminate the nature of the active sites in the confined Cat-*x*h catalysts. Figure 6a presents the in situ DRIFTS spectra of CO adsorbed at 30 °C on the reduced catalysts. With the decrease of the crystallite sizes of cobalt in the Cat-*x*h catalysts, the band of CO adsorbed in linear-type^35,36^ shifted from 2040 to 2030 cm^−1^, and the band of CO adsorbed in bridge-type^37,38^ also shifted from 1934 to 1921 cm^−1^. The red shift of the IR bands indicates the strengthened Co–C bond^39,40^. In addition, we also conducted the experiments of in situ DRIFTS with adsorption of syngas at 220 °C. In Supplementary Fig. 12, only linearly adsorbed CO band located at ~2055 cm^−1^ was observed^37^. As the crystallite size of cobalt decreases, the IR band exhibits the red shift, indicating an enhanced electron back-donation from the metallic *d* orbitals to the π* antibonding molecular orbital of CO. It is consistent with the result of DRIFTS with adsorption of CO at 30 °C. The smaller cobalt exposes more surface atoms with a lower coordination number, leading to an increased localization of the valence electrons. This localization causes an upward shift of the center of *d*-band, which will strengthen the bond between cobalt and adsorbate^9^.

In order to further understand the interaction between the adsorbed CO and the confined cobalt nanocrystals on the catalytic performance, we performed the CO-temperature programmed desorption/Mass spectroscopy (CO-TPD/MS) measurements over the reduced Cat-*x*h catalysts (Fig. 6b). The catalysts show one desorption peak of CO in the range of 270–430 °C. The desorption quantity and desorption temperature of CO increased with the decrease of the crystallite sizes of cobalt. According to the literatures^41,42^, the desorbed CO is generated from the recombination of the C* and O* species adsorbed on the metal sites. The findings from CO-TPD/MS indicate that the smaller cobalt crystallites can more strongly adsorb the C* species compared with the larger ones, resulting in the higher desorption temperature. It coincides with the results of in situ DRIFTS in Fig. 6a and Supplementary Fig. 12. Among the Cat-*x*h catalysts, the Cat-4h catalyst with the smallest cobalt crystallites possesses the lowest TOF value, because the strongest interaction between cobalt and adsorbate inhibits desorption of adsorbates therein. However, they present the similar apparent CO conversion (Table 2) because the Cat-4h catalyst with the smaller cobalt crystallites can expose more active sites, as listed in Table 1. Additionally, because of the high dispersion of cobalt, the large amount of the C* species exists on the Cat-4h catalyst.

The surface carbide mechanism^43–45^ is widely accepted to explain catalytic behaviors of FTS catalysts in Supplementary Fig. 13. In the carbide mechanism, direct CO dissociation occurs on the surface of active cobalt sites. The chemisorbed carbon (C*) species undergo hydrogenation to form CH_*x*_ (1 ≤ *x* *≤* 3) groups, and subsequently the CH_*x*_ groups act as monomers and chain initiators for chain growth. It is worthy to note that the CH_*x*_ coverage must be high enough to boost up chain growth^43,44^. As discussed in Fig. 6, the small-sized cobalt nanocrystals expose the more active sites (Table 1), which will create more reaction intermediates inside the confinement space to prolong their contact time, favoring the chain growth and the high selectivity towards heavy hydrocarbons. It can well explain our findings as opposed to the view that larger Co metallic crystallites produce heavier hydrocarbons^8,16,26,27^. Therefore, the differences in FTS product distribution observed in the Cat-*x*h catalysts are likely caused by the two factors including the intrinsic characteristic related to the crystallite size of cobalt and the confinement structure of the catalysts.

Supplementary Fig. 14 shows the thermal gravimetric (TG) profiles of the spent catalysts. The weight loss below 150 °C is attributed to removal of water, while that above 200 °C is ascribed to combustion of heavy FTS products^23^. In Supplementary Fig. 14, the heavier FTS products deposited on the surface of the spent Cat-*x*h catalysts with the smaller cobalt size, which is in agreement with the results of the FTS activity. It provides further evidence that the *α* value increases with the decreased crystallite size of the confined cobalt catalyst (Fig. 5b). Thus, the Cat-4h catalyst has the high selectivity towards the diesel-range products.

Supplementary Fig. 15 compares the TEM images of the spent catalysts and the corresponding crystallite size distributions. The crystallite sizes of cobalt had little change before and after the FTS reactions for the Cat-*x*h catalysts, but the cobalt species seriously aggregated on the Cat-IM catalysts. It indicates that the embedment of cobalt into silica can effectively inhibit its aggregation.

## Discussion

It is demonstrated that the embedment of cobalt into mesoporous silica support, likely water-melon seeds inside pulps, is a promising strategy to precisely control the crystallite size of the cobalt based FTS catalysts. The spatial confinement can effectively inhibit the aggregation of the cobalt nanocrystals during the FTS reactions, which avoids misunderstandings caused by size change, concerning the function of the crystallite sizes of cobalt on the selectivity of the products. Moreover, the contact time between the trapped reaction intermediates and active sites can be prolonged inside the confined space, further boosting up the growth of long chain hydrocarbons. We reveal that the crystallite size of the confined cobalt catalysts can significantly affect the catalytic behaviors in FTS. With the enlarged cobalt crystallites (*d*(Co^0^)_T_) from 7.2 nm of the Cat-4h to 11.4 nm of the Cat-12h, the TOF increased from 3.9 × 10^−2^ to 6.4 × 10^−2^ s^−1^, but the major product selectivity changed reversely from 66.2% of diesel-range hydrocarbons to 62.4% of gasoline-range hydrocarbons. Compared with the large size of cobalt, the small ones can strongly adsorb and capture the larger amount of the C* species, which guarantees the high enough CH_*x*_ coverage inside the confined space to stimulate chain growth by the carbide mechanism. Our results provide the new insights into the development of highly active and selective catalyst systems for the related industrial processes.

## Methods

### Synthesis of Co_3_O_4_ nanocrystal

Cobalt acetate of 1.17 g and TTAB of 4.26 g were added to a solution containing ethanol of 100 mL at 30 °C under vigorous stirring. Ammonia solution was utilized to control the pH value of the solution (pH = 8). Subsequently, the mixture was sealed in a 150 mL Teflon-lined autoclave and hydrothermally synthesized at 150 °C for *x* h (*x* = 4, 8, 12) to obtain the TTAB-capped Co_3_O_4_ nanocrystals. The corresponding Co_3_O_4_ nanocrystals were denoted as Co_3_O_4_-*x*h (*x* = 4, 8, 12).

### Co_3_O_4_ nanocrystals embedded into SiO_2_ supports

The suspension of the obtained TTAB-capped Co_3_O_4_ nanocrystals was added to ethanol of 185 mL and deionized water of 185 mL at 30 °C under vigorous stirring. Then, TTAB solution of 5.58 g containing TEOS of 20 mL was slowly added drop by drop to the above suspension. The suspension was kept stirring and refluxing at 60 °C for 15 h to form the Co_3_O_4_-TTAB-SiO_2_ nanocomposite.

### Thermal treatment

The obtained nanocomposite was retrieved by centrifugation and dried at 110 °C for 12 h. Then, the powder was calcined in air by raising its temperature to 400 °C with a ramping rate of 5 °C min^−1^ and holding at 400 °C for 4 h. The achieved catalyst was named as Cat-*x*h (*x* = 4, 8, 12), where ‘*x*h’ was the hydrothermal synthesis period of the Co_3_O_4_ nanocrystals.

### Synthesis of catalysts with different cobalt loading

Co_3_O_4_ nanocrystals were synthesized by the same method using hydrothermal synthesis period of 4 h, as mentioned above. The process of Co_3_O_4_ nanocrystals embedded into SiO_2_ supports was also similar to above described method, but the molar ratio of TEOS/Co was adjusted for controlling the embedment depth of cobalt crystallites. The TEOS/Co molar ratios of the Cat-4h-1, Cat-4h, and Cat-4h-2 were 2, 4, and 8, respectively. The final catalyst was obtained after drying and calcining under the same conditions.

For the purpose of comparison, we prepared reference catalysts by impregnation of as-synthesized Co_3_O_4_-*x*h nanocrystals over silica supports, which were named as Cat-IM-*x*h. Here, ‘IM’ standed for impregnation method and ‘*x*h’standed for hydrothermal periods of Co_3_O_4_ nanocrystals. And the other reference catalyst of the Cat-IM, was prepared by a conventional incipient-wetness impregnation method. Briefly, cobalt acetate was impregnated on the silica support. The heat treatment conditions including drying and calcining were the same as previously described. The mass fraction of the cobalt in all the catalyst was ~15% as determined by the inductively coupled plasma-mass spectroscopy (ICP-MS; Agilent 7700x).

### Transmission electron microscopy

TEM images were recorded on a JEM-2100F (JEOL Co.) microscope. The crystallite size distributions were calculated upon the measurement of 200 crystallites coming from several images taken at different positions on the TEM grid. The surface-averaged crystallite size was calculated from $d({\mathrm{Co}}^0)_{\mathrm{T}} = \frac{{\sum n_id_i^3}}{{\sum n_id_i^2}}$ with the standard deviation of $\sigma = \sqrt {\frac{{\Sigma {{n}}_i \cdot (d_i - d({\mathrm{Co}}^0)_{\mathrm{T}})^2}}{{\Sigma n_i}}}$, where *n*_*i*_ was the frequency of occurrence of each *d*_*i*_ size^7,46^.

### X-ray diffraction

XRD patterns were obtained on a Philips X’Pert Pro diffractometer using monochromatized Co Kα radiation (*λ* = 1.78897 Å), scanning 2*θ* from 20° to 80° with a scanning speed of 6° min^−1^ at 40 kV and 40 mA. Diffraction patterns were manually analyzed with the Joint Committee of Powder Diffraction Standard (JCPDS) card. The average crystallite size of the Co_3_O_4_ (*d*(Co_3_O_4_)_X_) was estimated from the Scherrer’s equation applied to the most intense (311) diffraction (2*θ* = 43.1°) using a shape factor *K* = 0.9^47^. The mean crystallite size of metallic cobalt (*d*(Co^0^)_X_) was then obtained from the corresponding crystallite size of Co_3_O_4_ by applying the molar volume correction: $d({\mathrm{Co}}^0)_{\mathrm{X}} = \frac{3}{4}d({\mathrm{Co}}_3{\mathrm{O}}_4)_{\mathrm{X}}$.

### X-ray absorption spectroscopy

X-ray absorption measurements were carried out at 14W1 beamline in Shanghai Synchrotron Radiation Facility (SSRF) and 1W1B beamline in Beijing Synchrotron Radiation Facility (BSRF). The X-ray Absorption Fine Structure (XAFS) data were collected in the transmission mode through a Si (111) double crystal monochromator. Co_3_O_4_ and Co foil were used as reference compounds for Extended X-ray Absorption Fine Structures (EXAFS) analysis. The ex-situ spectra of Co K-edge of the Cat-*x*h catalysts and references were recorded at room temperature (RT) in transmission mode. For the collection of in situ XANES spectra at RT, a mixture gas composing of 10 vol. % H_2_/N_2_ was introduced into the in situ sample chamber allowing heating to 400 °C for 10 h, and then the cell was cooled down to RT. We calibrated the monochromator by setting the first inflection point of the K-edge spectrum of the Co foil at 7709 eV.

### Nitrogen adsorption measurements

The nitrogen adsorption-desorption isotherms were determined on a Quantachrome Quadrasorb SI apparatus at −197 °C. The specific surface areas were obtained in a relative pressure range from 0.05 to 0.30 and were calculated using the Brunaue-Emmett-Teller (BET) method. The total pore volume was calculated from the amount of N_2_ vapor adsorbed at a relative pressure of 0.99.

### Chemical adsorption experiment

Temperature programmed reduction (TPR), H_2_-O_2_ titration and temperature programmed desorption (TPD) experiments were carried out with a TPDRO apparatus (TP-5080, Tianjin Xianquan Co., Ltd) equipped with a thermal conductivity detector (TCD). TPR measurement was performed in a flow of 8 vol. % H_2_/N_2_ (30 mL min^−1^) at a heating rate of 10 °C min^−1^ from RT to 900 °C.

The amounts of surface metal atoms of the cobalt catalysts were measured by H_2_-chemisorption experiments. The sample of 100 mg was reduced at 400 °C for 10 h in H_2_ flow, and then evacuated at 400 °C for 1 h in N_2_ flow prior to measurement of H_2_ titration at 100 °C. Dispersion percentage (*D*%) was calculated according to the equation $D\% = \frac{{{\mathrm{1}}{\mathrm{.179}}X}}{{Wf}}$, where *X* was the total H_2_ uptake in micromoles per gram of catalyst, *W* was the weight percentage of cobalt, and *f* was the reduction degree calculated from O_2_-titration. Reduction degrees of the reduced catalysts were determined by O_2_-titration at 400 °C in a flow of helium. The reaction of the metallic cobalt species with oxygen was considered to form Co_3_O_4_. The particle sizes of cobalt (*d*(Co^0^)_H_), were estimated from the total amount of chemisorbed H_2_, assuming an atomic ratio of H/Co = 1, and a hemispherical crystallite geometry with a surface atomic density of 14.6 atoms nm^−2^. Thus, $d({\mathrm{Co}}^0)_{\mathrm{H}} = \frac{{{\mathrm{96}}}}{{D\% }}$.

CO-TPD/MS experiments were carried out to study the interaction between cobalt crystallite surfaces and carbon monoxide. The sample of 100 mg was reduced at 400 °C for 10 h in H_2_ flow in a quartz reactor, and then evacuated at 400 °C for 1 h in He flow prior to measurement of CO adsorption at RT. The sample was purged by He flow (30 mL min^−1^) at RT for 1 h, and then was heated from RT to 800 °C (10 ^o^C min^−1^). The signal of CO m/z = 28 was monitored with MS (HPR-20 QIC, Hiden Analytical Ltd.).

### In situ X-ray photoelectron spectroscopy

In situ XPS spectra were recorded on a PHI 5000 Versa Probe with an aluminum anode (Al Kα = 1486.6 eV) operating at 25 W. In the case of pretreated sample, the as-prepared catalysts were placed in the introduction chamber (IC), and then were transferred to the reaction chamber (RC) by a transfer rod. After degassing the RC, the samples were reduced by H_2_ at 400 °C. As the temperature was cooled down from 400 °C to RT, the samples were transferred back to IC, and were degassed again to remove the adsorbed H_2_. Subsequently, the samples were transferred to the main chamber (MC) for XPS detection with an accuracy of ± 0.1 eV.

### In situ infrared spectroscopy

In situ diffuse reflectance infrared Fourier transform spectroscopy (DRIFTS) spectra were collected on a Nexus FT-IR spectrometer equipped with a MCT detector at the liquid nitrogen temperature and a diffuse reflectance attachment in the range of 650–4000 cm^−1^. The sample (30–40 mg) was placed in an infrared cell with a ZnSe window and reduced for 10 h at atmospheric pressure in a hydrogen stream at 400 °C. Subsequently, the system was cooled down to RT or 220 °C, and the background spectra were recorded. After introduction of carbon monoxide or syngas for 0.5 h, the catalyst was purged with helium at RT or hydrogen at 220 °C for 0.5 h. All spectra were recorded with a resolution of 2 cm^−1^ and accumulation of 32 scans.

### Catalytic tests

The FTS tests were carried out in a stainless steel fixed-bed reactor (i.d. 8 mm) with a catalyst loading of 0.5 g (pellet size: 0.25-0.42 mm). Prior to reaction, the catalysts were reduced in pure hydrogen at 400 °C for 10 h. After the reduction, unless otherwise specified, the catalytic tests were carried out at 220 °C and 2 MPa in syngas (CO/H_2_ = 1/2, W/F = 5.1 g_cat_ h mol^−1^). The reaction pressure was maintained by a back-pressure regulator. The thermocouple directly contacted the catalyst bed, and by moving thermocouples, we checked that no heat spot was detected during the FT reactions. Additionally, besides syngas, the feeding gas contained 1.5 vol.% nitrogen, which was used as an internal standard for calculating the carbon monoxide conversion. To avoid possible condensation of the reaction products, the temperature of the whole pipelines from reactor to gas chromatography (GC) for product analysis was kept at 220 °C during the catalytic tests. Gaseous reaction products were analyzed on-line by GC. Hydrocarbons were separated on a HP-PONA capillary column of GC and analyzed with a flame-ionization detector (FID). The analysis of H_2_, N_2_, CO, CH_4_, and CO_2_ was performed with a TDX-01 packed column of GC and a TCD. The analysis of lighter paraffins and olefins (C_1_–C_5_) was carried out with an HP-PLOT/Q capillary column of GC. CO conversion (%) and product selectivity (%) were defined in supplementary information. Carbon balances were better than 90 % for all of the tests in this work. The chain growth probability (*α*) was evaluated from the slope of straight lines using C_14_–C_21_ products determined by equation $Wn = n \cdot \alpha ^{n - 1} \cdot (1 - \alpha )^2$. All the catalytic results of the catalysts were obtained after no less than 20 h on stream.

### Data availability

The data that support the findings of this work are available within the article and its Supplementary Information files. All other relevant data supporting the findings of this study are available from the corresponding author on reasonable request.

## Electronic supplementary material

Supplementary Information
