# Supplementary material for: Confined small-sized cobalt catalysts stimulate carbon-chain growth reversely by modifying ASF law of Fischer–Tropsch synthesis
Source: Nat Commun. 2018 Aug 14;9:3250. doi: 10.1038/s41467-018-05755-8 (PMC6092428; doi:10.1038/s41467-018-05755-8)
Supplement: Supplementary file 1 — Supplementary Information [file 41467_2018_5755_MOESM1_ESM.pdf]

Supplementary Information

Confined small-sized cobalt catalysts stimulate carbon-chain growth reversely by modifying ASF law of Fischer-Tropsch synthesis

Cheng et al.

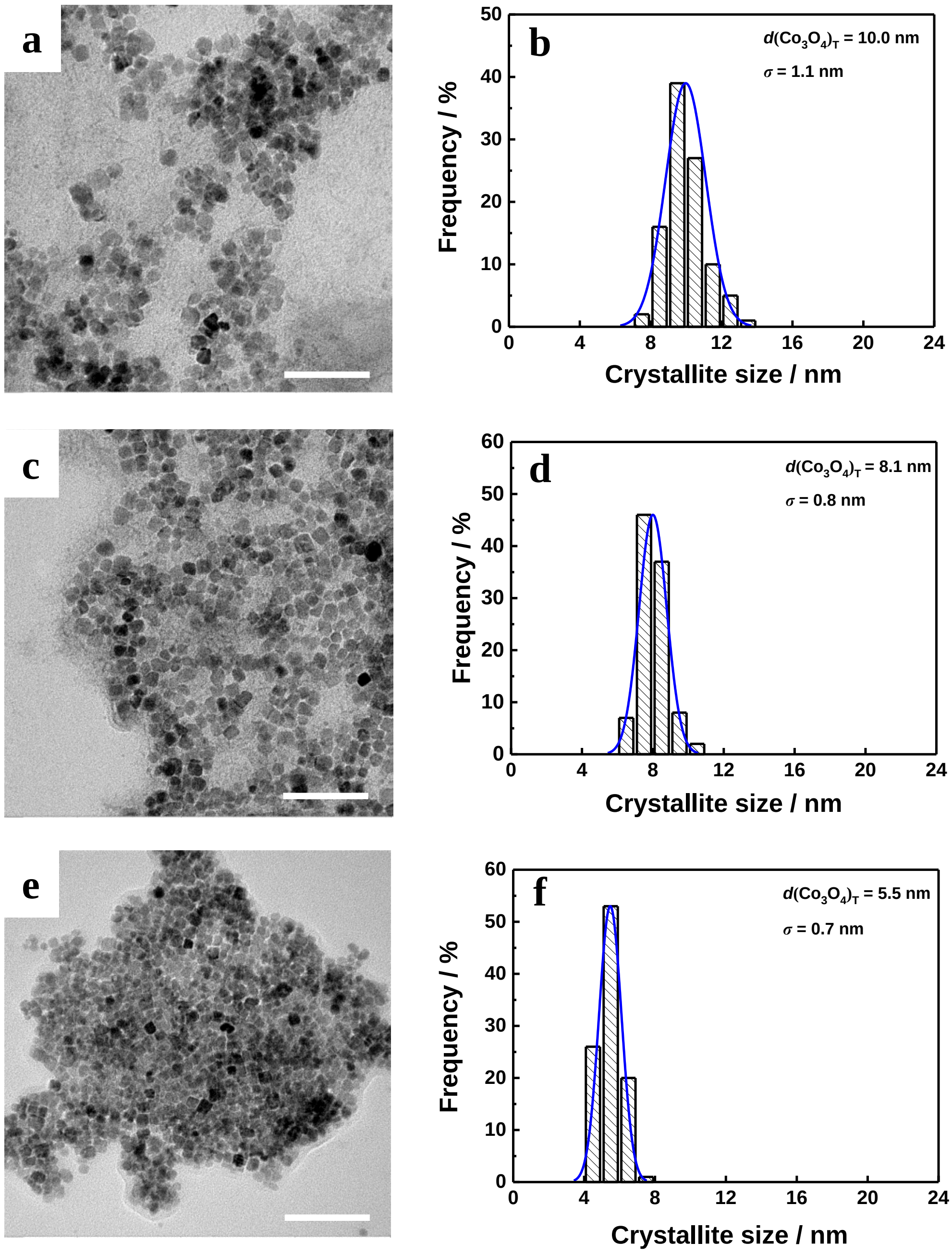

Supplementary Figure 1 | Controllable cobalt crystallite sizes by varying hydrothermal duration (12 h, 8 h, and 4 h). **a**, **c**, **e** TEM images of the TTAB-capped Co_3O_4 (Co_3O_4-*x*h) nanocrystals with the different hydrothermal periods: **a** 12 h, **c** 8 h, and **e** 4 h. **b**, **d**, **f** Corresponding crystallite size distribution histograms. Scale bars: 50 nm.

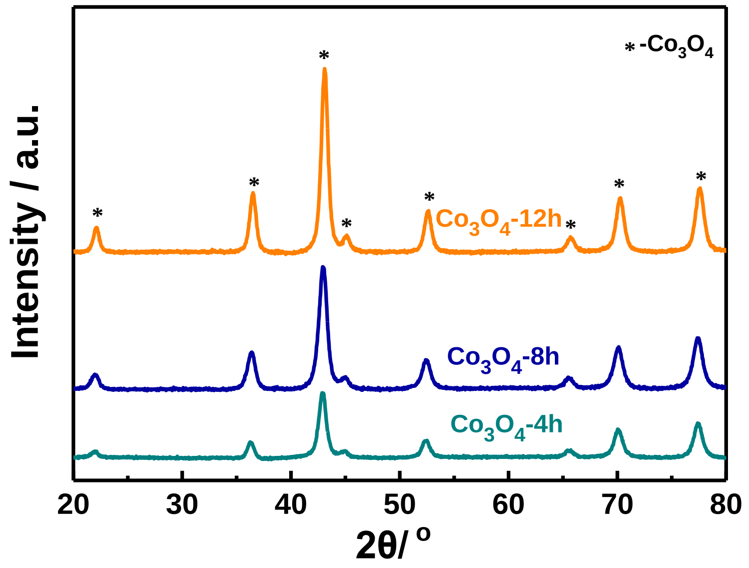

Supplementary Figure 2 | XRD patterns of the Co_3O_4-xh nanocrystals. All diffraction peaks can be indexed to the Co_3O_4 phase (JCPDS 74-2120) and are marked with filled asterisk.

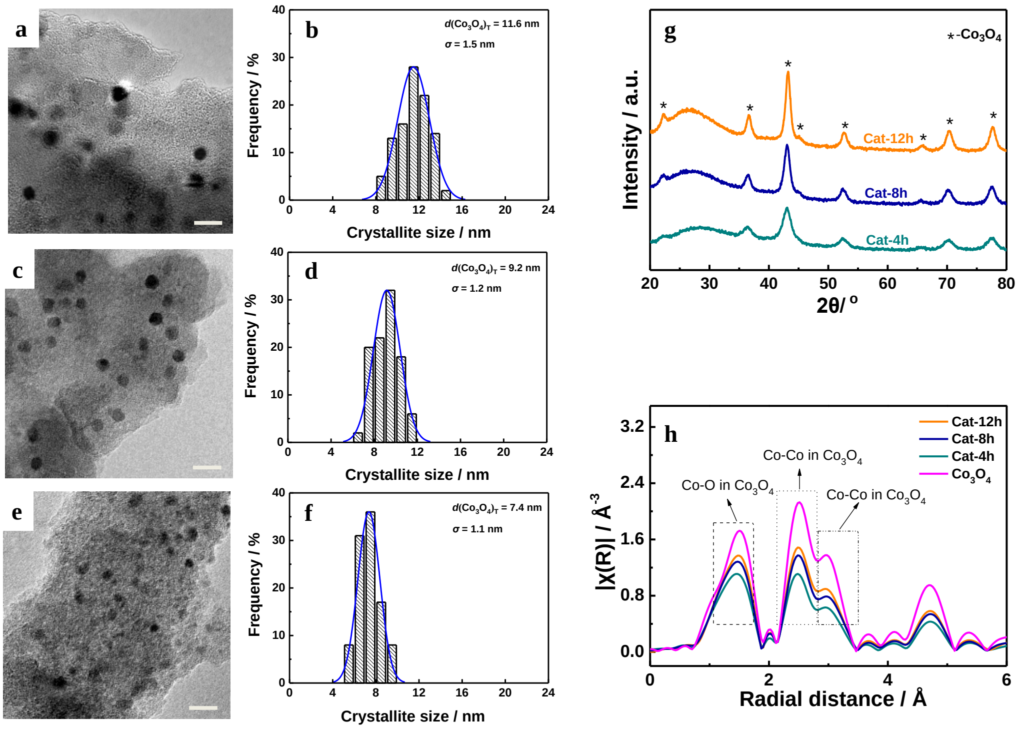

Supplementary Figure 3 | Structural characterizations of the Cat-*x*h catalysts. a, **b** Cat-12h. **c**, **d** Cat-8h. **e**, **f** Cat-4h. **a**, **c**, **e** TEM images of the Cat-*x*h catalysts. **b**, **d**, **f** Corresponding crystallite size distribution histograms. The Cat-*x*h catalysts present a narrow size distribution. The size of Co_3O_4 nanocrystals increases with the prolonged hydrothermal duration. **g** XRD patterns of the Cat-*x*h catalysts. All diffraction peaks can be indexed to the Co_3O_4 phase (JCPDS 74-2120) and are marked with filled asterisk. **h** RDFs of the Cat-*x*h catalysts and the reference Co_3O_4. The cobalt species exist in the form of Co_3O_4. The intensities of the peaks are obviously different, which implies the coordination numbers of the cobalt species in the sequence of Cat-12h > Cat-8h > Cat-4h[1,2]. That is, the change of the crystallites sizes of the Co_3O_4 follows the sequence of Cat-12h > Cat-8h > Cat-4h[3,4]. These results are in good agreement with the above results of XRD. Scale bars: 20 nm.

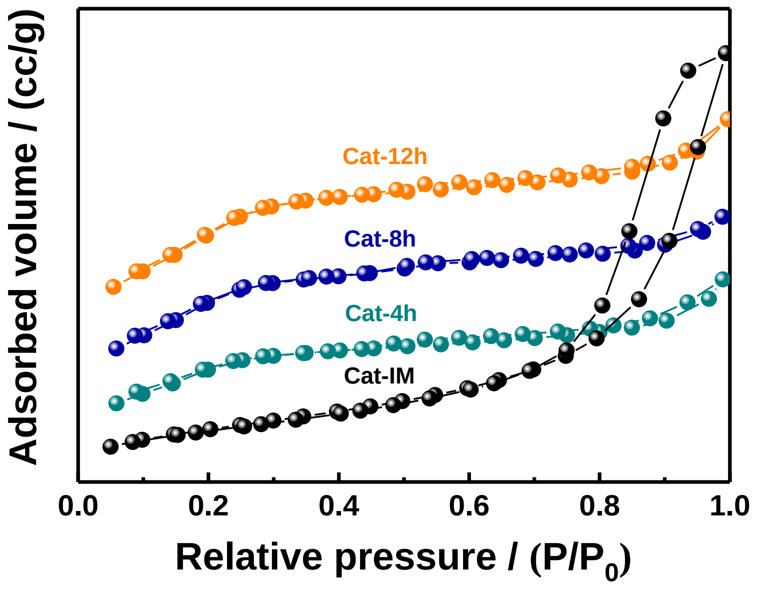

Supplementary Figure 4 | Nitrogen adsorption-desorption isotherms of the catalysts.

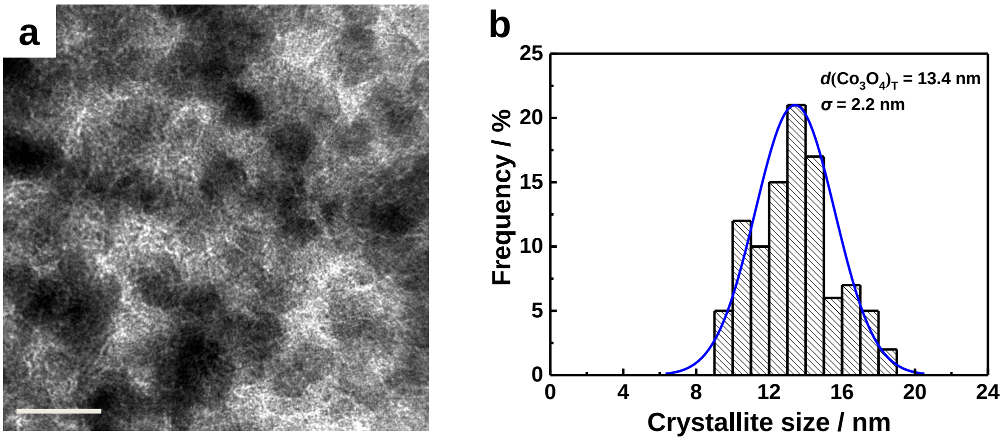

S**upplementary Figure 5 | Co_3O_4 crystallites of the Cat-IM catalyst with a broad size distribution. a** TEM image of the Cat-IM catalyst. The Co_3O_4 crystallites are poorly dispersed on the surface of the Cat-IM catalyst. **b** Corresponding crystallite size distribution histograms. The Co_3O_4 crystallites present a board size distribution. Scale bars: 20 nm.

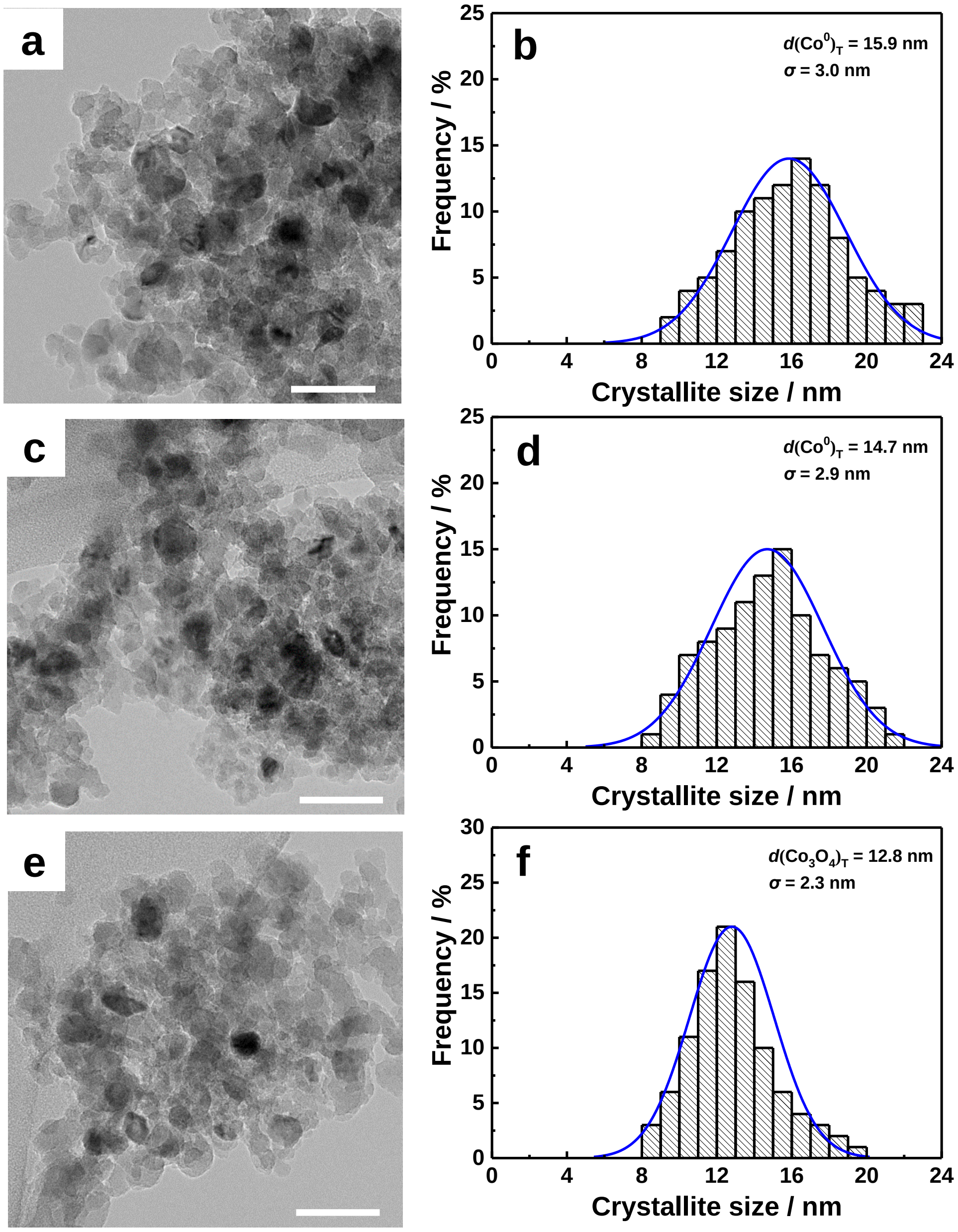

Supplementary Figure 6 | Metallic cobalt crystallites of the reduced Cat-IM-*x*h catalysts with a broad size distribution. **a**, **c**, **e** TEM images of the reduced Cat-IM-*x*h catalysts. **b**, **d**, **f** Corresponding crystallite size distribution histograms. **a**, **b** Cat-IM-12h. **c**, **d** Cat-IM-8h. **e**, **f** Cat-IM-4h. Scale bars: 50 nm.

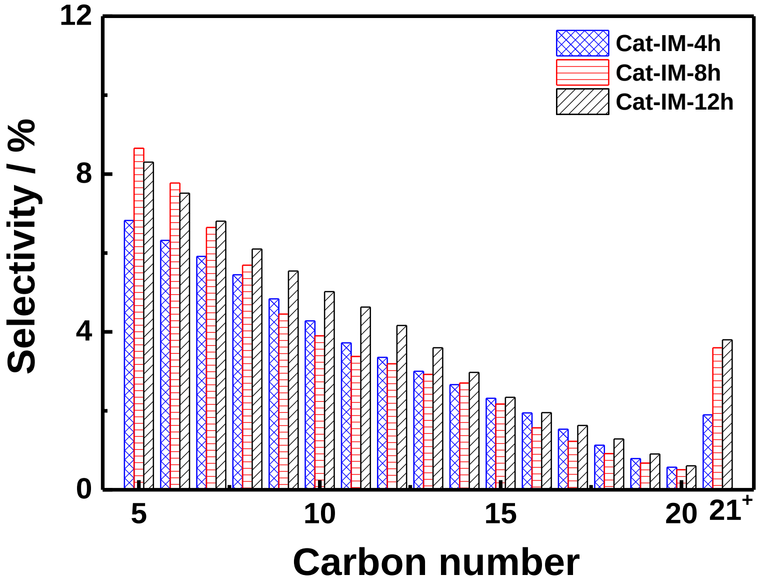

Supplementary Figure 7 | Distribution of the C_5^+ products of the Cat-IM-*x*h catalysts.

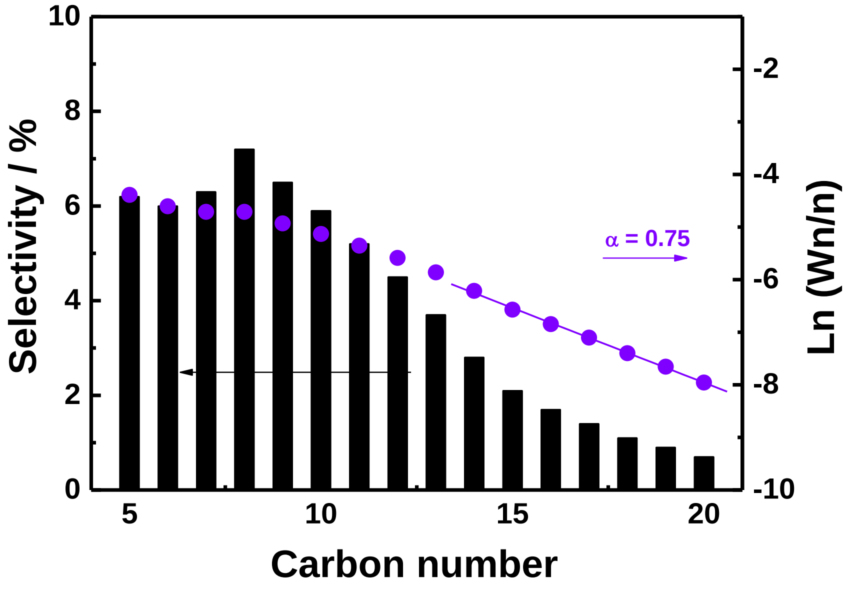

Supplementary Figure 8 | Distribution of C_5^+ products and ASF distribution for the Cat-IM catalyst.

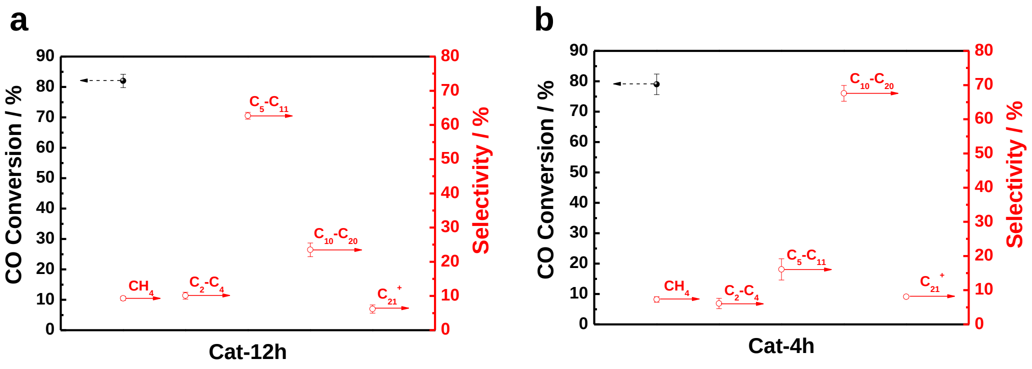

Supplementary Figure 9 | Repeatability of the catalytic performance of the catalysts. a Cat-12h, and **b** Cat-4h. Reaction conditions: P=2 MPa, T=220 °C, W/F=5.1 g h mol^{-1}, CO/H_2 =1/2. The black ball in **a** and **b** denotes CO conversion. The red circle in **a** and **b** denotes product selectivity. The experiments were repeated for three times. Error bars indicate s.d. (n = 4).

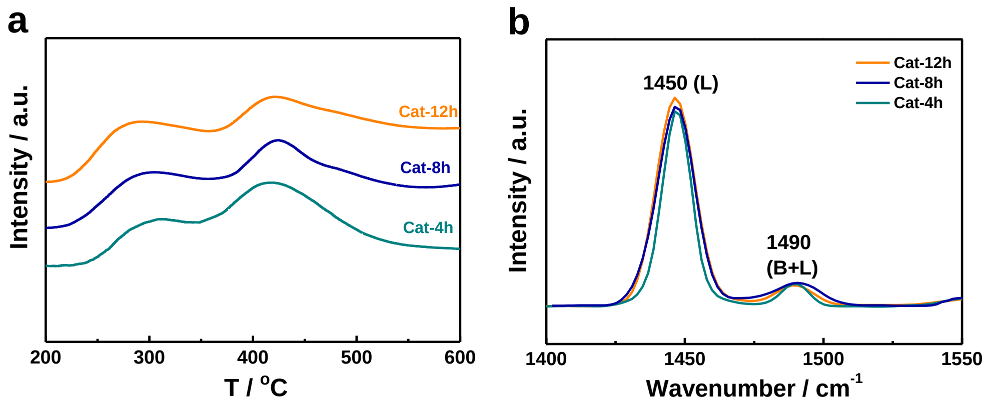

Supplementary Figure 10 | Characterization of surface acidity. a NH_3-TPD profiles for the Cat-xh catalysts, and **b** Fourier transform infrared spectra of pyridine adsorption for the Cat-*x*h catalysts.

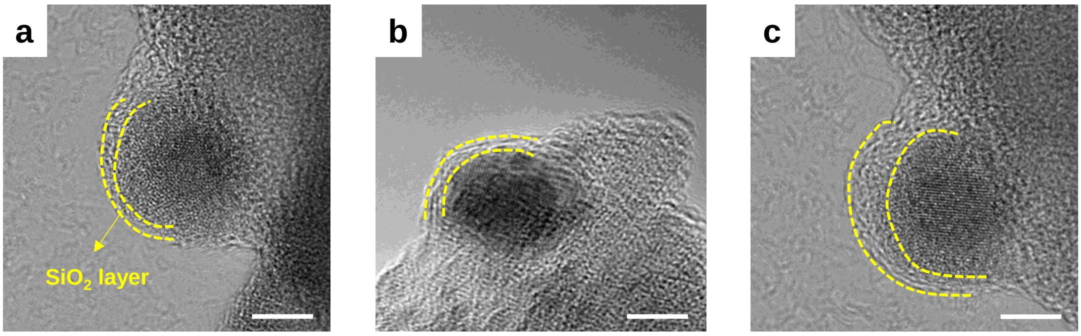

Supplementary Figure 11 | TEM images of the reduced catalysts. a Cat-4h-1, **b** Cat-4h, and **c** Cat-4h-2. Scale bars: 5 nm.

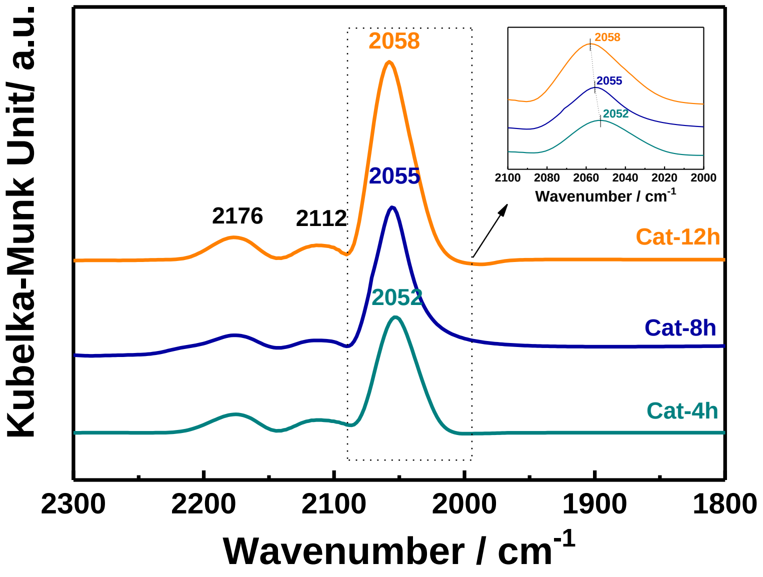

Supplementary Figure 12 | In situ DRIFTS spectra with adsorption of syngas of the reduced Cat-*x*h catalysts at 220 °C.

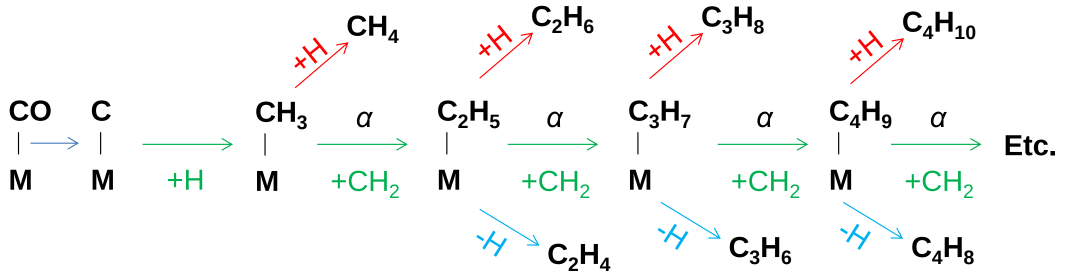

Supplementary Figure 13 | Surface carbide mechanism for FTS[5,6].

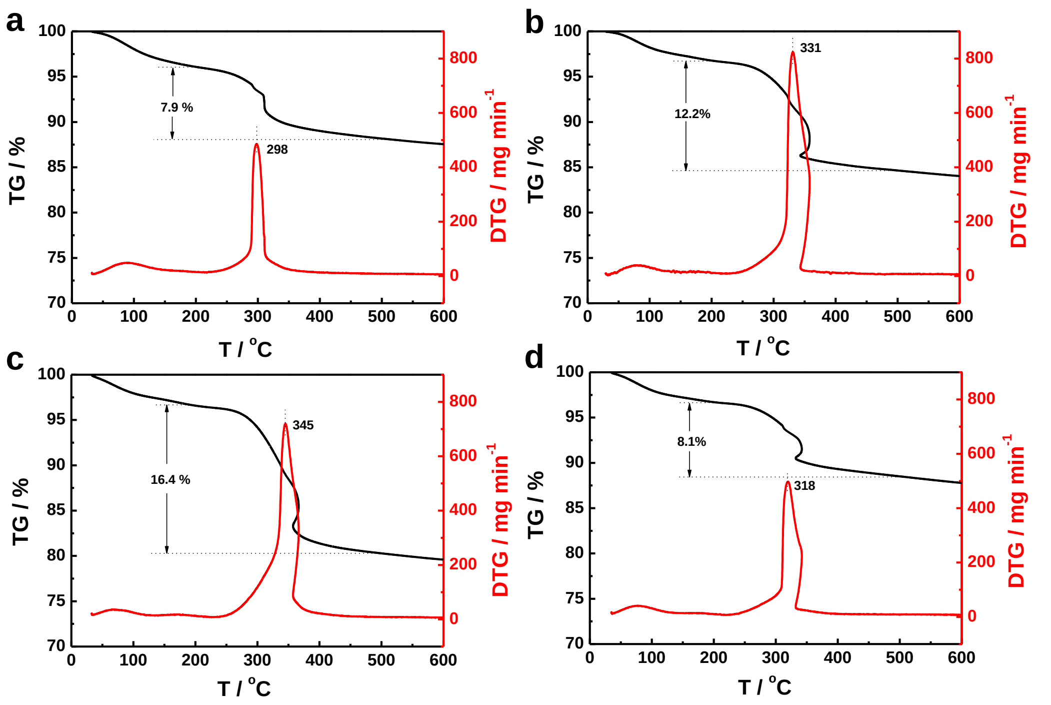

Supplementary Figure 14 | Thermal gravimetric profiles of the spent catalysts. a Cat-12h, **b** Cat-8h, **c** Cat-4h, and **d** Cat-IM.

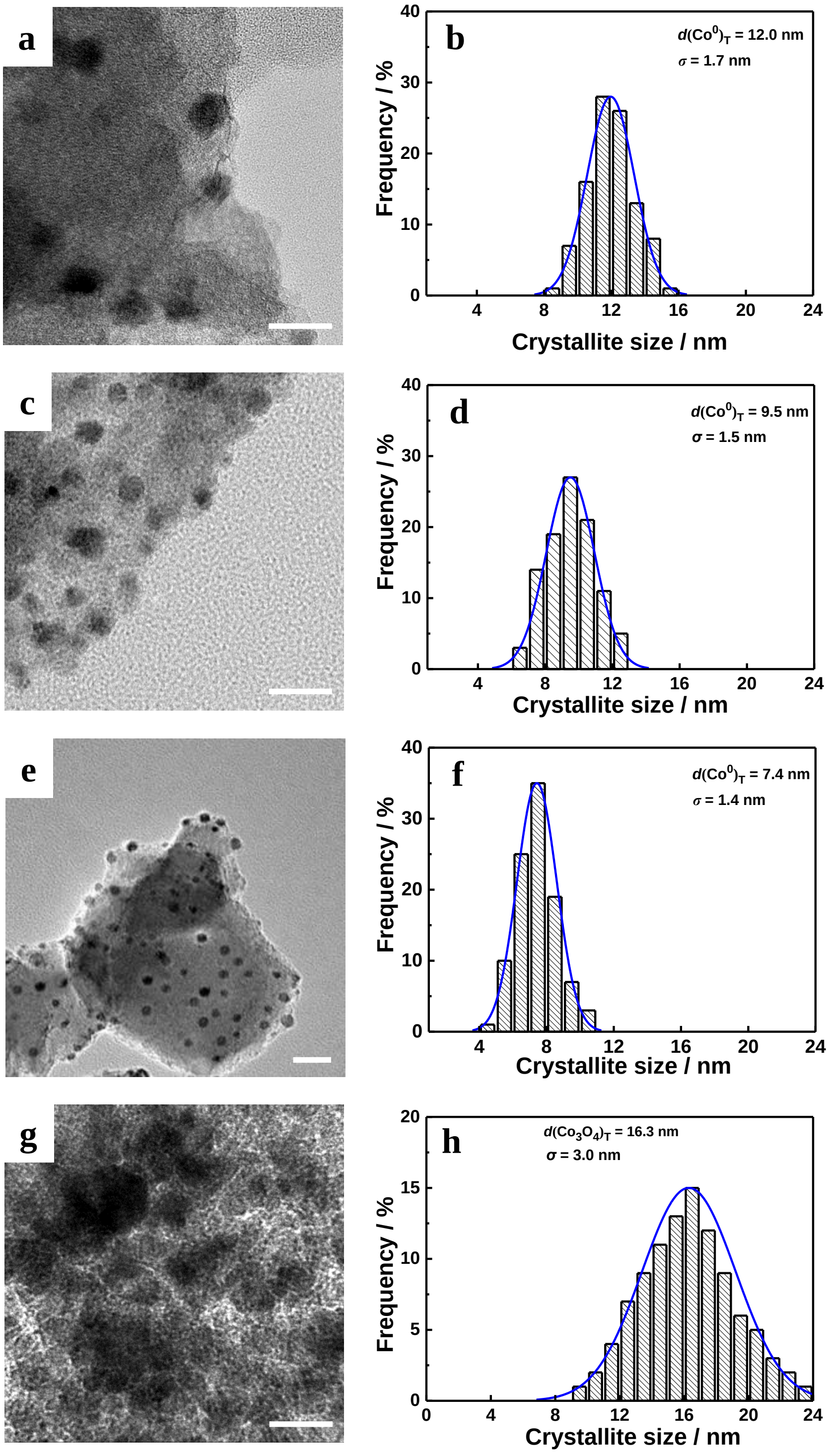

Supplementary Figure 15 | TEM images and corresponding crystallite size distributions of the spent catalysts. **a**, **c**, **e**, **g** TEM images of the spent catalysts, and **b**, **d**, **f**, **h** corresponding crystallite size distribution histograms. **a**, **b** Cat-12h. **c**, **d** Cat-8h. **e**, **f** Cat-4h. **g**, **h** Cat-IM. Scale bars: 20 nm.

Supplementary Table 1 | EXAFS parameters of coordination shell for the Cat-*x*h catalysts and the Co_3O_4.

Samples	First shell				Second shell			
	**Bond**	***R* / Å**	***N***	**σ^2 / Å^2**	**Bond**	***R* / Å**	***N***	**σ^2 / Å^2**
Co_3O_4	Co-O	1.92	4.0	0.003	Co-Co	2.85	6.0	0.003
Cat-12h	Co-O	1.92	3.4	0.004	Co-Co	2.86	4.5	0.005
Cat-8h	Co-O	1.92	2.6	0.005	Co-Co	2.84	4.0	0.001
Cat-4h	Co-O	1.92	1.3	0.008	Co-Co	2.84	3.4	0.004

Supplementary Table 2 │ EXAFS parameters of coordination shell for the reduced Cat-*x*h catalysts and the Co-foil.

Samples	First Shell			
	**Bond**	***R* / Å**	***N***	**σ^2 / Å^2**
Co-foil	Co-Co	2.49	12.0	0.006
Cat-12h	Co-Co	2.50	9.3	0.007
Cat-8h	Co-Co	2.50	8.1	0.007
Cat-4h	Co-Co	2.50	7.3	0.006

Supplementary Table 3 │ H_2-TPR results of the catalysts.

Catalysts	Peak I		Peak II		Peak III		Peak IV		Peak III / ΣPeak / %
	T / °C	Area	T / °C	Area	T / °C	Area	T / °C	Area	
Cat-12h	267	1239	356	3922	496	7805	709	5688	41.8
Cat-8h	252	1048	342	4253	522	9430	716	5418	46.8
Cat-4h	237	3207	323	2170	510	10199	708	4957	49.7
Cat-IM	211	799	342	7880	453	4580	778	9828	19.8

Supplementary Table 4 │ FTS activity data of the Cat-IM-*x*h catalysts. [a]

Catalysts	CO Conversion (%)	TOF (s^{-1})	Selectivity (%)					
			CH_4	C_2-C_4	C_5^+	C_5-C_{11}	C_{10}-C_{20}	C_{21}^+
Cat-IM-4h	44.6	0.029	21.8	21.7	56.5	37.3	25.3	1.9
Cat-IM-8h	46.2	0.035	21.5	18.4	60.1	40.5	23.2	3.6
Cat-IM-12h	46.8	0.042	21.3	15.3	63.4	43.9	29.1	3.8

[a] Reaction condition: P=2 MPa, T=220 °C, W/F=5.1 g_{cat} h mol^{-1}, CO/H_2=1/2.

Supplementary Table 5 | FTS activity data of the Cat-12h catalyst repeated for three times.[a]

repetitions	CO Conversion / %	Selectivity / %					
		CH_4	C_2-C_4	C_5^+	C_5-C_{11}	C_{10}-C_{20}	C_{21}^+
1st	80.6	8.7	11.3	80.0	62.4	23.7	5.7
2nd	85.1	10.1	8.9	81.0	61.7	25.8	7.4
3rd	80.2	9.0	10.0	81.0	64.1	20.9	5.5

[a] **Reaction condition:** P=2 MPa, T=220 °C, W/F=5.1 g_{cat} h mol^{-1}, CO/H_2=1/2.

Supplementary Table 6 | FTS activity data of the Cat-4h catalyst repeated for three times.[a]

repetitions	CO Conversion / %	Selectivity / %					
		CH_4	C_2-C_4	C_5^+	C_5-C_{11}	C_{10}-C_{20}	C_{21}^+
1st	77.0	8.0	7.8	84.2	15.6	66.2	7.8
2nd	83.7	7.6	4.1	88.3	20.1	65.7	8.5
3rd	76.2	6.2	6.5	87.3	12.5	70.9	7.9

[a] **Reaction condition:** P=2 MPa, T=220 °C, W/F=5.1 g_{cat} h mol^{-1}, CO/H_2=1/2.

Supplementary Table 7 | FTS activity data of the catalysts with the different Co loading. [a]

Catalysts	Catalyst weight (g)	Cobalt loading (wt. %)	CO Conversion (%)	TOF (s^{-1})	Selectivity (%)					
					CH_4	C_2-C_4	C_5^+	C_5-C_{11}	C_{10}-C_{20}	C_{21}^+
Cat-4h-1	0.25	29.8	68.4	0.036	8.5	8.8	82.7	16.7	71.5	13.2
Cat-4h	0.5	15.3	77.0	0.038	8.0	7.8	84.2	15.6	66.2	7.8
Cat-4h-2	0.9	8.3	80.3	0.039	9.3	5.8	84.9	32.5	60.4	3.0

[a] Reaction condition: P=2 MPa, T=220 °C, W/F=0.78 g_{Co} h mol^{-1}, CO/H_2 =1/2.

Supplementary Table 7 shows the FTS activity data of the embedded catalysts with the different Co loading. The local H_2/CO ratio in the confined space is higher than that in the inlet gas because of the smaller size of H_2 molecule than CO. It will slightly inhibit the growth of carbon chain on cobalt during FTS. Thus, the Cat-4h-1 catalyst with a thinner embedment depth, i.e. a lower silica content, exhibits a slightly higher selectivity towards the diesel fraction and C_{21}^+. It should be noted here that all of these catalysts show the high selectivity towards the diesel fraction because they have the same size of cobalt crystallites.

Supplementary Methods

Chemicals and reagents. Cobalt acetate (99.9 %), tetraethyl orthosilicate (99.99 %), ammonia (30 %) and amorphous silica (99.8 %) were purchased from Shanghai aladdin Biochemical Technology Co., Ltd. Tetradecyltrimethlammonium bromide (99 %) and ethanol (AR) were purchased from Macklin Biochemical Technology Co., Ltd.

Catalytic characterization. NH_3-TPD experiments were carried out on a TPDRO instrument (TP-5080; Tianjin Xianquan Co., Ltd). The sample of 100 mg loaded into a quartz reactor was heated at 200 °C for 1 h in N_2 flow (30 mL min^{-1}). After the sample was cooled to 150 °C, 10 vol. % NH_3/N_2 flow (30 mL min^{-1}) was introduced for 0.5 h to adsorb ammonia. Before the NH_3-TPD experiment, the physisorbed ammonia on the sample was purged by He flow (30 mL min^{-1}) at 150 °C for 1 h, and then the sample was heated from 150 to 800 °C (10 °C min^{-1}). The desorbed NH_3 was analyzed by a thermal conductivity detector (TCD).

Fourier transform infrared spectroscopy (FT-IR) of pyridine adsorption was conducted in a high vacuum system to determine the type and the quantity of the acid sites on the samples. The sample of 30 mg was pressed into a disk with a diameter of 10 mm and placed in an IR cell with CaF_2 windows. After pretreatment under vacuum at 300 °C for 1 h, the sample was cooled to 50 °C, and then pyridine was adsorbed at 50 °C on the sample for a sufficient time. FT-IR spectra were recorded after gaseous or weakly adsorbed pyridine molecules were removed by evacuation at 150 °C.

Thermal analysis was carried out with the samples after no less than 20 h FTS reactions on a DTG-60 (Shimadzu) to investigate FTS products which formed on the samples. It was

implemented in an air flow of 50 mL min^{-1}. The temperature increased from room temperature to 600 °C at a rate of 10 °C min^{-1}.

Catalytic activity. The conversion of CO (%) was calculated by the difference in CO/N_2 ratio between chromatograms taken at the gas inlet before reaction and chromatograms taken at the gas outlet during FTS reactions (Supplementary Equation 1).

$$X_{CO} = \frac{\frac{CO_{inlet}}{N_{2,inlet}} - \frac{CO_{outlet}}{N_{2,outlet}}}{\frac{CO_{inlet}}{N_{2,inlet}}} \times 100 \quad (1)$$

X_{CO} was the conversion of CO. CO_{inlet} and $N_{2,\text{inlet}}$ were the peak areas of the corresponding gases in the TCD chromatograms of the syngas inlet before FTS reactions. CO_{outlet} and $N_{2,\text{outlet}}$ were the peak areas of the outlet composition during FTS reactions.

The carbon selectivity (%) of each product was defined as below (Supplementary Equation 2):

$$S_{Cn} = \frac{nF_{Cn}}{\sum_{n=1}^{N} nF_{Cn}} \times 100 \quad (2)$$

S_{Cn} was the carbon selectivity toward a product with n carbon atoms. F_{Cn} indicated the molar flow toward a product with n carbon atoms.

The TOF value (s^{-1}) was calculated from the following expression (Supplementary Equation 3):

$$\text{TOF} = \frac{moles\ of\ reacted\ CO}{(moles\ of\ Co_{surface}) \times reaction\ time} = \frac{\frac{velocity\ of\ raw\ gases}{1000 \times 22.4} \times precent\ of\ CO \times converion\ of\ CO}{weight\ of\ catalysts \times content\ of\ Co \times \frac{dispersion}{relative\ moler\ mass} \times 60} \quad (3)$$

Supplementary References

1. Ernst, B. *et al.* Study on a cobalt silica catalyst during reduction and Fischer-Tropsch reaction: In situ EXAFS compared to XPS and XRD. *Catal. Today* **39**, 329-341 (1998).

2. Xu, K. *et al.* e-Iron carbide as a low-temperature Fischer-Tropsch synthesis catalyst. *Nat. Commun.* **5**, 5783-5790 (2014).

3. Bian, G. Z. *et al.* Investigations on the structural changes of two Co/SiO_2 catalysts by performing Fischer-Tropsch synthesis. *Appl. Catal. A* **252**, 251-260 (2013).

4. Jacobs, G. *et al.* Fischer-Tropsch synthesis: Temperature programmed EXAFS/XANES investigation of the influence of support type, cobalt loading, and noble metal promoter addition to the reduction behavior of cobalt oxide particles. *Appl. Catal. A* **333**, 177-191 (2007).

5. Torres Galvis, H. M. & de Jong, K. P. Catalysts for production of lower olefins from synthesis gas: A review. *ACS Catal.* **3**, 2130-2149 (2013).

6. Ojeda, M. *et al*. CO activation pathways and the mechanism of Fischer-Tropsch synthesis. *J. Catal.* **272**, 287-297 (2010).
